# Progress of Surgery

**Published:** 1890-05-24

**Authors:** 


### SURGERY.

### PROGRESS OF SURGERY.

There is a very large volume, published 1730, entitled "A General System of Surgery," by Dr. Laurence Heister, of the University of Delmstadt. It professes to be a complete system of the art of surgery, and is illustrated by numerous woodcuts, both of the instruments then in use and of the manner of performing the different operations. One large plate is peculiarly interesting. It represents the manner of removing the leg below the knee-joint. The surgeons are dressed in wigs and tails, laced coats, and knee-breeches. The patient is seated in a large arm-chair; one assistant holds the limb above the knee, a second holds the foot, and the operator is represented as sawing through the bone. We are told that the "antient surgeons," in amputating the foot at the tarsus or metatarsus, used a large chisel and mallet, and sometimes a large pair of cutting pincers, and then treated and healed the wound with balsams. But Heister in his voluminous work improves upon the practice of the "antient surgeons." He advises that the dressings of the stump be made with a pledgit of lint, with small linen compresses upon the ends of the divided arteries, previously secured by ligature. But, he adds, that, in his own opinion, it would be better to apply no compresses to the arteries or bone. Although such full directions are given throughout Heister's book, we do not find therein any reference made to the rate of mortality occurring after operations as then performed. It was stated, long before even the days of Heister, that a surgeon skilled in his art was "more than armies to the public weal." If that were the case in remote times, the surgeon of the present day must be worth more than many armies to the public weal. For, perhaps, there is scarcely any department of science and art in which greater advances have been made during recent periods.

Mr. Frederick Page, surgeon to the Royal Infirmary, Newcastle-on-Tyne, has reprinted from the "Northumberland and Durham Medical Society's Transactions," his report on the results of major operations treated antiseptically in the Royal Infirmary during the eleven years and nine months ending December 31st, 1889. Mr. Page includes the following under the heading major operations, viz., double amputations, hip joint, thigh, knee joint, leg, ankle joint, shoulder joint, arm, forearm, and wrist. The total number of amputations during the period noted above is 536, with 42 deaths, or a mortality of 7·8 per cent. 201 were operated upon for injury with 25 deaths, or 12·4 per cent. 335 amputations were for disease, and 17 patients died, or 5 per cent. During the year 1889 it was found necessary to perform 54 major amputations. On two occasions a double operation was performed. Five patients died and all the others recovered. The mortality is, therefore, 9·6 per cent. Twenty more of the operations were for disease, and two patients died. No death followed an amputation performed for disease in the years 1888 and 1887, so that during the last three years there were 101 consecutive amputations for disease with only two deaths. Mr. Page observes "a better result can hardly be looked for." Evidently Mr. Page attributes a considerable proportion of success to antiseptic treatment. Recently there has been rather a disposition to place less confidence in antiseptics, and more in perfect cleanliness, which, however, is practically antiseptic treatment. But as compared with surgery, in the days of Heister, there are other reasons besides antiseptics which tend to success. These are the use of anæsthetics, improved instruments and ligatures, improved methods of applying the latter, greater skill on the part of the operator, and not delaying operation till the case is hopeless.

